# Bioremediation of Historically Chlorimuron-Ethyl-Contaminated Soil by Co-Culture Chlorimuron-Ethyl-Degrading Bacteria Combined with the Spent Mushroom Substrate

**DOI:** 10.3390/microorganisms8030369

**Published:** 2020-03-05

**Authors:** Hailian Zang, Wanjun Liu, Yi Cheng, Hailan Wang, Xuejiao An, Shanshan Sun, Yue Wang, Ning Hou, Chunyu Cui, Chunyan Li

**Affiliations:** 1 College of Resources and Environment, Northeast Agricultural University, Harbin 150030, Heilongjiang, China; 10303188@163.com (H.Z.); liuwanjun@yeah.net (W.L.); hailan_218@163.com (H.W.); sss907777522@163.com (S.S.); wangyuekun@neau.edu.cn (Y.W.); houning571@163.com (N.H.);; 2 College of Science, China Agricultural University, Beijing 100083, China; chengyi58918@163.com; 3 College of Bioscience and Bioengineering, Jiangxi Agricultural University, Nanchang 330045, China; mds1999@163.com

**Keywords:** chlorimuron-ethyl, co-culture bacteria, contaminated soil, bioremediation, spent mushroom substrate, microbial community structure

## Abstract

In this study, a novel chlorimuron-ethyl-degrading *Pleurotus eryngiu*-SMS-CB was successfully constructed for remediation of soil historically contaminated with chlorimuron-ethyl. The *P. eryngiu*-SMS-CB was prepared using efficient chlorimuron-ethyl-degrading cocultured bacteria, *Rhodococcus* sp. D310-1 and *Enterobacter* sp. D310-5, with spent mushroom substrate (SMS, a type of agricultural waste containing laccase) of *Pleurotus eryngiu* as a carrier. The chlorimuron-ethyl degradation efficiency in historically chlorimuron-ethyl-contaminated soil reached 93.1% at the end of 80 days of treatment with the *P. eryngiu*-SMS-CB. Although the *P. eryngiu*-SMS-CB altered the microbial community structure at the beginning of the 80 days, the bacterial population slowly recovered after 180 days; thus, the *P. eryngiu*-SMS-CB does not have an excessive effect on the long-term microbial community structure of the soil. Pot experiments indicated that contaminated soil remediation with *P. eryngiu*-SMS-CB reduced the toxic effects of chlorimuron-ethyl on wheat. This paper is the first to attempt to use chlorimuron-ethyl-degrading bacterial strains adhering to *P. eryngiu*-SMS to remediate historically chlorimuron-ethyl-contaminated soil, and the microbial community structure and *P. eryngiu*-SMS-CB activity in chlorimuron-ethyl-contaminated soil were traced in situ to evaluate the long-term effects of this remediation.

## 1. Introduction

Chlorimuron-ethyl (ethyl 2-(4-chloro-6-methoxypyrimidin-2-ylcarbamoylsulfamoyl) benzoate) is a long-term residual (2–3 years) sulfonylurea herbicide used to control important broadleaved weeds in soybean crops, and it is characterized by high efficiency, low toxicity and a broad spectrum [1]. However, long-term and excessive application damages subsequent susceptible crops, affects the soil microbial community and decreases soil enzyme activities, and its continued presence in soil impacts rotational crops [2,3]. Thus, it is necessary to find an effective method to decrease or remove the toxicity of chlorimuron-ethyl residue in contaminated soil.

Bioremediation using chlorimuron-ethyl-degrading bacteria offers an efficient, economic, and safe method of eliminating chlorimuron-ethyl contamination, and biological methods are an eco-friendly approach compared to chemical and physical methods [4,5]. Although the biodegradation of chlorimuron-ethyl has been revealed in previous studies, chlorimuron-ethyl-degrading strains that can be applied to remediate soil contaminated with chlorimuron-ethyl are lacking. Yang et al. used a solution containing the chlorimuron-ethyl-degrading strain CHL1 to remediate chlorimuron-ethyl-contaminated soil and investigated the lifetime of CHL1 and changes in the abundance of fungi and bacteria over the bioremediation period [6]. Unfortunately, degrading bacteria do not live long in liquid media because such media are easily contaminated; additionally, the preservation of these bacteria in liquid media is unsatisfactory [7]. 

The main obstacle to bioremediation is the inability of the introduced microorganisms to tolerate the harsh environmental conditions in the field [8]. Therefore, exploring a solid carrier to which a mass of live bacteria can adhere and that can maintain the vitality of bacteria by providing a protective surface and a microhabitat that preserves the bacteria is essential [9,10]. Notably, a form of agricultural waste, spent mushroom substrate (SMS), which is a soil-like material that remains after a crop of mushrooms is harvested [2], can be employed as a carrier to reduce costs and efficiently utilize waste. SMS is environmentally friendly and rich in organic matter; it can improve soil fertility and has beneficial effects on soil biochemical properties and microbiological parameters [11,12]. Thus, involving comprehensive consideration of environmental benefits, economic benefits, waste recycling and other factors, the use of SMS has attracted considerable attention. The surface features of SMS, which include many cavities and enzymes (including laccase), may promote the adsorption or degradation of organic and inorganic pollutants [13]. For example, Marínbenito et al. reported that pesticides were absorbed by SMS, and Singh et al. found that SMS contains laccase [14,15]. Zeng et al. concluded that laccase has high potential for treating industrial wastewater containing the herbicide isoproturon [16]. However, previous studies have not focused on chlorimuron-ethyl-degrading bacterial strains adhering to laccase-containing SMS to remediate historically chlorimuron-ethyl-contaminated soil. In our previous study, a mixture of the high-efficiency chlorimuron-ethyl-degrading bacteria *Rhodococcus* sp. D310-1 and *Enterobacter* sp. D310-5 were used to ferment and prepare a chlorimuron-ethyl-degrading bacterial co-culture [9]. The chlorimuron-ethyl degradation efficiency reached 80.02% after 60 days in the greenhouse (in soil contaminated with chlorimuron-ethyl at 20 mg kg^−1^), demonstrating that the application of the bacterial chlorimuron-ethyl-degrading agent promoted the degradation of chlorimuron-ethyl in the soil and reduced its phytotoxicity toward wheat. Nevertheless, there were still some deficiencies in the preparation of a chlorimuron-ethyl-degrading bacterial coculture for remediating chlorimuron-ethyl-contaminated soil, as follows: (1) Previous study had focused on the application of bacteria to soil with simulated short-term pollution by chlorimuron-ethyl. As a consequence, the remediation effect could not be completely equivalent to that on historically contaminated soil. (2) The microbial community was evaluated by Illumina MiSeq sequencing after only 60 days of remediation of the simulated chlorimuron-ethyl-contaminated soil; this period was relatively short and could not accurately reflect the effects of co-culture bacteria on the microbial community in historically chlorimuron-ethyl-contaminated soil. Compared to the study of Li (2017), this article was improved in the following ways: First, historically chlorimuron-ethyl-contaminated soil was remediated by the chlorimuron-ethyl-degrading *P. eryngiu*-SMS-CB. Second, the *P. eryngiu*-SMS-CB was produced based on spent mushroom substrate (SMS, agricultural waste) as a carrier, which contains many enzymes, especially laccase. Third, the biodegradation of historically chlorimuron-ethyl-contaminated soil was monitored for 180 days, and the bacterial community structure and the genus-level abundances of co-culture bacteria were analyzed to evaluate the effects of the *P. eryngiu*-SMS-CB. 

In this study, the highly efficient chlorimuron-ethyl-degrading bacteria *Rhodococcus* sp. D310-1 and *Enterobacter* sp. D310-5 were used with *Pleurotus eryngiu* SMS as a carrier for preparation of a chlorimuron-ethyl-degrading *P. eryngiu*-SMS-CB. The response surface method (RSM) was used to optimize the culture and preparation conditions of the *P. eryngiu*-SMS-CB to improve the chlorimuron-ethyl degradation efficiency. Moreover, historically chlorimuron-ethyl-contaminated soil (with a chlorimuron-ethyl concentration of 19.2 mg kg^−1^) was remediated by the *P. eryngiu*-SMS-CB. The composition of the soil bacterial community was assessed by Illumina MiSeq sequencing, and then the changes in bacterial diversity, variance, and genus-level abundances during the bioremediation processes (80 d and 180 d) were analyzed for assessing the interaction between *P. eryngiu*-SMS-CB and other bacteria in situ. Changes in the plant height, root length and fresh weight of wheat grown in soil with different levels of chlorimuron-ethyl stress were determined, and changes in the activity of superoxide dismutase (SOD) and the content of soluble proteins (SPs) in wheat were examined to evaluate the long-term effects of the remediation. This study is the first to attempt to use the highly efficient chlorimuron-ethyl-degrading co-culture bacteria adhering to the agricultural waste material-SMS to remediate historically chlorimuron-ethyl-contaminated soil. The objective of our study is to offer a novel strategy for the bioremediation of chlorimuron-ethyl-contaminated soil.

## 2. Materials and methods

### 2.1. Bacterial Strains

*Rhodococcus* sp. D310-1 and *Enterobacter* sp. D310-5 were isolated from activated sludge samples obtained from a factory producing sulfonylurea herbicides in China and then stored in our laboratory: *Rhodococcus* sp. D310-1 (GenBank accession number, GU138102.1; the maximum biodegradation efficiency of 88.95% was obtained with a substrate concentration of 100 mg·L^−1^, a pH value of 6.0, an inoculum size of 1.94% and a temperature of 28 °C) [17] and *Enterobacter* sp. D310-5 (GenBank accession number: KU204704; the maximum biodegradation efficiency of 87.6% was obtained with a substrate concentration of 100 mg·L^−1^, a pH value of 6.6, an inoculum size of 2.01% and a temperature of 30 °C) [18].

### 2.2. Chemicals and Media

Chlorimuron-ethyl (98.7% purity) was supplied by Jiangsu Institute of Ecomones Co., Ltd., China. All chemicals and analytical reagents used in this study were provided by Tianjin Kemiou Chemical Reagent Co., Ltd., China. Laccase (CAS:80498-15-3) was purchased from Beijing Solarsoft Technology Co., Ltd., China.

The composition of the minimal salt media (MSM) was as follows: CaSO_4_ 0.04 g·L^−1^, K_2_HPO_4_ 0.1 g·L^−1^, NaCl 0.1 g·L^−1^, FeSO_4_·7H_2_O 0.001 g·L^−1^, MgSO_4_·7H_2_O 0.2 g·L^−1^ and (NH_4_)_2_SO_4_ 0.1 g·L^−1^, pH 6.0-6.5.

### 2.3. Soil Samples and SMS

Soil samples were collected from the experimental farm of the Jiamusi Branch of the Heilongjiang Academy of Agricultural Sciences in Heilongjiang Province, Northeast China (46°82′ N, 130°4′ E), in which chlorimuron-ethyl has been used as an herbicide in soybean crops with corn crop rotation for 10 years. During corn planting periods, the herbicide used was acetochlor. The soil samples were sieved through a 2-mm mesh screen for physicochemical characterization. The physical and chemical properties of the soil were as follows: 96.7 mg kg^−1^ total nitrogen (N), 46.3 mg kg^−1^ total phosphorus (P), 419 mg kg^−1^ total potassium (K), water-holding capacity 4.51%, and pH 6.62. The concentration of chlorimuron-ethyl in the soil was 19.2 mg kg^−1^. 

*SMS of Pleurotus eryngiu and Pleurotus ostreatus was sourced from Bingrong Bio-Technology Co., Ltd. in Heilongjiang Province, China.*

### 2.4. The Chlorimuron-Ethyl Extraction Assay

The chlorimuron-ethyl extraction assay was performed according to Li et al. [17], and the detailed information is described in the supplementary material. The samples were filtered through a 0.22-μm millex-gp pes filter for hplc analysis [8].

### 2.5. HPLC Instrumentation and Conditions

The chlorimuron-ethyl concentration was determined via HPLC (Waters, MA, USA) according to the method referenced by Zang et al. [19]. The detailed information is described in the supplementary material.

### 2.6. Effects of Laccase Crude Extract from SMS on the Degradation of Chlorimuron-Ethyl and Carrier Selection

*Pleurotus eryngiu* and *Pleurotus ostreatus* are the two most commonly cultivated species of edible mushrooms in north China; they have large cultivation volumes and generate a large amount of SMS. Considering the actual application, we chose the *Pleurotus eryngiu* SMS and *Pleurotus ostreatus* SMS as alternative carriers. The laccase activity and the ability to degrade chlorimuron-ethyl of the same volume of laccase crude extract from SMS were used as the indices to determine which SMS was more suitable to be a carrier. The extraction of laccase crude extract and the enzyme activity assay are described in the supplementary material. One milliliter of laccase crude extract from *P. eryngiu* SMS or *P. ostreatus* SMS was added to MSM (100 mL) supplemented with 20 mg L^−1^ chlorimuron-ethyl and incubated at 27 °C for 7 d (pH 6.5, 160 rpm), and then the concentration of residual chlorimuron-ethyl was measured. The chlorimuron-ethyl degradation efficiency of laccase (CAS:80498-15-3) was determined to ensure that the laccase activity was similar to that in the crude extract of the final selected SMS.

### 2.7. Degradation of Chlorimuron-Ethyl by Sterilized P. eryngiu-SMS and Unsterilized P. eryngiu-SMS

The chlorimuron-ethyl degradation efficiency of sterilized *P. eryngiu*-SMS (S-e-SMS) and unsterilized *P. eryngiu*-SMS was tested. The sterilized and unsterilized *P. eryngiu*-SMS were added to MSM supplemented with 20 mg L^−1^ chlorimuron-ethyl and cultured at 27 °C for 7 d, and then the amount of chlorimuron-ethyl residue was determined.

### 2.8. Preparation of the P. eryngiu-SMS-CB

*P. eryngiu*-SMS was inoculated with co-culture bacteria (*Rhodococcus* sp. D310-1 and *Enterobacter* sp. D310-5 (1:1)) to prepare the chlorimuron-ethyl-degrading *P. eryngiu*-SMS-CB. During the preparation process, D310-1 and D310-5 were cultivated and collected, and then the concentration of the bacteria was adjusted with a phosphate buffer to OD_600nm_ = 2.0 ± 0.1. Finally, they were inoculated into the fermentation medium with 2% inoculum, respectively [20]. Detailed information related to the preparation of the co-culture bacteria and the *P. eryngiu*-SMS-CB are provided in the supplementary material. The preparation conditions for the *P. eryngiu*-SMS-CB were optimized using the RSM based on the single-factor test [21]. The *P. eryngiu*-SMS-CB and the empty carrier were observed by scanning electron microscopy (SEM). To eliminate the influence of pH on chlorimuron-ethyl, the effect of pH on chlorimuron-ethyl degradation was assessed first (detailed information in the supplementary material, and the results are shown in Figure S1).

### 2.9. Degradation of Chlorimuron-Ethyl by P. eryngiu-SMS, Co-Culture Bacteria and P. eryngiu-SMS-CB

The chlorimuron-ethyl degradation efficiencies of dried *P. eryngiu*-SMS (drying temperature 38 °C), co-culture bacteria (D310-1 and D310-5 were adjusted with a phosphate buffer to OD_600nm_ = 2.0 ± 0.1, inoculated into the fermentation medium with 2% inoculum, respectively, and co-cultured to the logarithmic phase), and *P. eryngiu*-SMS-CB were tested. The *P. eryngiu*-SMS (5 g), co-culture bacteria (OD = 1.92, 3 mL), and *P. eryngiu*-SMS-CB (5 g) were added to 100 mL MSM supplemented with 20 mg L^−1^ chlorimuron-ethyl and cultured at 27 °C for 7 d, and the chlorimuron-ethyl level was quantified.

### 2.10. Remediation of Chlorimuron-Ethyl-Contaminated Soil

The *P. eryngiu*-SMS-CB dosage added to the soil was optimized in a greenhouse (the temperature ranged from 17 to 26 °C with an average relative air humidity of 45% to 65%). Different *P. eryngiu*-SMS-CB dosages (0.1 g kg^−1^, 0.5 g kg^−1^, 1.0 g kg^−1^, 1.5 g kg^−1^ and 2.0 g kg^−1^) were added to the historically chlorimuron-ethyl-contaminated soil. The chlorimuron-ethyl degradation efficiency was determined by HPLC after 30 days as described in Section 2.5. All of the treatments were replicated three times to minimize experimental error. The soil collected and prepared as described in Section 2.3 was split into four groups. Four treatments were set up in triplicate: real soil (RS, as control group); real soil inoculated with *P. eryngiu*-SMS (*P. eryngiu*-SMS); real soil inoculated with co-cultured bacteria (CB); and real soil inoculated with *P. eryngiu*-SMS-CB (*P. eryngiu*-SMS-CB). Sterile water was added to the soil of each groups every 2 days to maintain the moisture content. Soil samples were taken at specific time intervals (10, 20, 30, 40, 50, 60, 70 and 80 d) for chlorimuron-ethyl residue quantification. A high-throughput sequencing method (MiSeq platform) was used by Sangon Biotech Co., Ltd. (Shanghai, China) to examine the bacterial relative abundance and diversity in the samples at 80 days and 180 days [22,23].

### 2.11. Plant Culture

After 80 days, wheat was planted in the real soil (RS, as control group), real soil inoculated with *P. eryngiu*-SMS (*P. eryngiu*-SMS), real soil inoculated with co-cultured bacteria (CB), and real soil inoculated with *P. eryngiu*-SMS-CB (*P. eryngiu*-SMS-CB) plots. The plant height and root length were measured directly with a ruler at 10, 20, 30, and 40 days. The fresh weight was determined using an electric balance (AL204, Shanghai, China).

### 2.12. Determination of SOD Activity and SP

Superoxide dismutase (SOD) was determined by the method of Huang et al. [24]. Briefly, samples (0.5 g fresh leaves) were ground in liquid nitrogen and extracted with 2 mL of 50 mM sodium phosphate buffer (pH 7.8). The samples were centrifuged at 4 °C, 10,000 r min^−1^ for 10 min and the supernatant was collected. All extracting experiments were carried out in the ice bath. SOD activity was determined by estimating its ability of inhibiting the photochemical reduction of nitroblue tetrazolium (NBT). Then the change in absorbance of the solution was recorded for 10 min at 560 nm. The amount of enzyme that inhibited the 50% NBT reduction was defined as one unit of SOD activity. The soluble protein (SP) content was determined by the dye-binding method, and the results are expressed as mg g^−1^ [25].

### 2.13. Data Analysis and Statistics

All values reported are the mean (±SD) of three replicates. Two-way and three-way analyses of variance (ANOVAs) were carried out using SPSS 17.0 (SPSS, Chicago, Illinois, USA) to determine significant effects (*p* < 0.05*, *p* < 0.01**, or *p* < 0.001***). The experimental design and data analysis were conducted using the statistical software Design Expert V. 8.0.6 (Stat-Ease, Inc. Minneapolis, USA).

## 3. Results and Discussion

### 3.1. Degradation of Chlorimuron-Ethyl by Crude Extracts Containing Laccase from SMS and Carrier Selection

**Figure 1:**
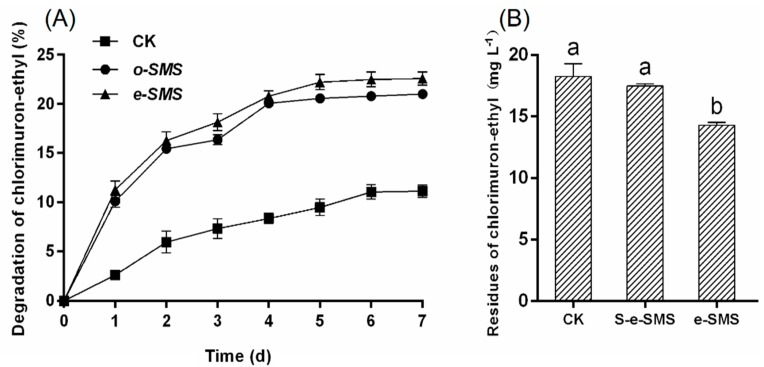
Selection of the carrier for the chlorimuron-ethyl-degrading co-culture bacteria. (**A**) Levels of chlorimuron-ethyl after treatment with crude extracts containing laccase from spent mushroom substrate (SMS). e-SMS: laccase crude extract from *P. eryngiu* SMS; o-SMS: laccase crude extract from *P. ostreatus* SMS. (**B**) Degradation of chlorimuron-ethyl by sterilized *P. eryngiu* SMS and unsterilized *P. eryngiu* SMS. S-e-SMS: sterilized *P. eryngiu* SMS; e-SMS: unsterilized *P. eryngiu* SMS. The bars represent the SDs of assays performed in triplicate.

Zeng reported that isoproturon could be degraded by laccase [16]. However, laccase applications are hindered by its high production costs and ready inactivation under industrial conditions [26,27,28]. Thus, a more inexpensive and viable alternative needs to be developed. Singh et al. found that SMS contains laccase; therefore, it is important to determine whether chlorimuron-ethyl could be degraded by laccase from SMS [15]. Figure 1A shows the degradation of chlorimuron-ethyl by the laccase crude extract from *P. eryngiu* SMS and *P. ostreatus* SMS. The chlorimuron-ethyl-degrading capability of the laccase crude extract from *P. ostreatus* SMS was less than that of the corresponding extract from *P. eryngiu* SMS, and the remaining chlorimuron-ethyl levels were determined to be 15.8 mg L^−1^ and 15.4 mg L^−1^, respectively, after 7 days. These values were significantly different from that of the control group at 17.8 mg L^−1^ (*p* < 0.01). There was no significant difference in the residual chlorimuron-ethyl between the laccase crude extract from the *P. eryngiu*-SMS (15.4 mg L^−1^) and laccase (CAS:80498-15-3) (15.1 mg L^−1^), which indicated that the laccase crude extract and the laccase (CAS:80498-15-3) had a similar effect on chlorimuron-ethyl degradation resulting from similar enzyme activity (24.8 ± 0.10 U g^−1^, 25.1 ± 0.10 U g^−1^). The above results indicated that chlorimuron-ethyl was degraded by the laccase crude extract from SMS. Thus, the laccase activity and the ability to degrade chlorimuron-ethyl of the same volume of laccase crude extract from SMS were comprehensively considered, and *P. eryngiu*-SMS was chosen as a carrier for the preparation of the chlorimuron-ethyl-degrading *P. eryngiu*-SMS-CB.

### 3.2. Degradation of Chlorimuron-Ethyl by Sterilized P. eryngiu-SMS and Unsterilized P. eryngiu-SMS

The efficiencies of chlorimuron-ethyl degradation by sterilized *P. eryngiu*-SMS (S-e-SMS) and unsterilized *P. eryngiu*-SMS are shown in Figure 1B. The concentration of residual chlorimuron-ethyl was 14.3 mg L^−1^, which was obviously less than that of S-e-SMS (17.5 mg L^−1^). The surface features of the SMS include many cavities and enzymes (including laccase), which likely promote the degradation of chlorimuron-ethyl [13]. Compared with the control group, S-e-SMS had a certain chlorimuron-ethyl adsorption effect but no significant effect on chlorimuron-ethyl residue, indicating that the degradation of chlorimuron-ethyl depends on enzymes from *P. eryngiu*-SMS.

### 3.3. Preparation of the P. eryngiu-SMS-CB

The *P. eryngiu* SMS was chosen as a carrier for the preparation of chlorimuron-ethyl-degrading *P. eryngiu*-SMS-CB. Based on the single-factor test (Figure S2), RSM was used to optimize the inoculation dose, culture time and culture temperature of the chlorimuron-ethyl-degrading *P. eryngiu*-SMS-CB. The model and Design Expert 8.0 (the ANOVA for the second-order polynomial model is shown in Table S1, and three-dimensional response surface plots is shown in Figure S3) were used to predict the best values of the three key factors as follows: inoculum quantity of 2.99 mL, culture temperature of 35.7 °C and culture time of 2.87 days. Under the above conditions, the highest degradation efficiency of *P. eryngiu*-SMS-CB was 94.4%. The results of optimization of the preparation conditions are provided in the supplementary material.

The carrier was inoculated with the fermentation liquid to produce the *P. eryngiu*-SMS-CB. The carrier morphology was characterized by SEM before and after inoculation. The surface of *P. eryngiu*-SMS was rough and contained many lacunae, providing a larger surface area for adsorption. Compared with that of the empty carrier *P. eryngiu*-SMS, a large number of bacteria were enriched on the carrier surface of the *P. eryngiu*-SMS-CB. SMS clearly provided the inoculated bacteria with a protective microhabitat (Figure S4).

### 3.4. Degradation of Chlorimuron-Ethyl by SMS, Co-Culture Bacteria and P. eryngiu-SMS-CB

The degradation of chlorimuron-ethyl by the *P. eryngiu*-SMS, co-culture bacteria (CB), and *P. eryngiu*-SMS-CB was determined using chlorimuron-ethyl residue concentrations of 1.11 mg L^−1^ (*P. eryngiu*-SMS-CB), 4.01 mg L^−1^ (CB), 14.3 mg L^−1^ (*P. eryngiu*-SMS) and 17.6 mg L^−1^ (control groups, CK). The chlorimuron-ethyl levels were significantly different among the CB, *P. eryngiu*-SMS-CB, *P. eryngiu*-SMS and CK. The residual chlorimuron-ethyl of the *P. eryngiu*-SMS-CB group was obviously less than that of the CB, *P. eryngiu*-SMS and CK groups, and the results showed that the *P. eryngiu*-SMS-CB group had a high chlorimuron-ethyl-degrading efficiency.

### 3.5. Remediation of Chlorimuron-Ethyl-Contaminated Soil by the P. eryngiu-SMS-CB

#### 3.5.1. Effects of the *P. eryngiu*-SMS-CB dosage on Chlorimuron-Ethyl Degradation in Soil

The chlorimuron-ethyl degradation efficiencies were 45.3%, 56.2%, 58.2%, 57.9% and 58.1% when the dose was 0.1 g·kg^−1^, 0.5 g·kg^−1^, 1.0 g·kg^−1^, 1.5 g·kg^−1^ and 2.0 g·kg^−1^, respectively. Analysis of the *P. eryngiu*-SMS-CB dose added to soil on the chlorimuron-ethyl degradation efficiency showed that the maximum degradation efficiency was obtained when the dose was 1.0 g kg^−1^. From an economic point of view, 1.0 g kg^−1^ was chosen as the *P. eryngiu*-SMS-CB dosage.

#### 3.5.2. Remediation of Chlorimuron-Ethyl-Contaminated Soil

**Figure 2:**
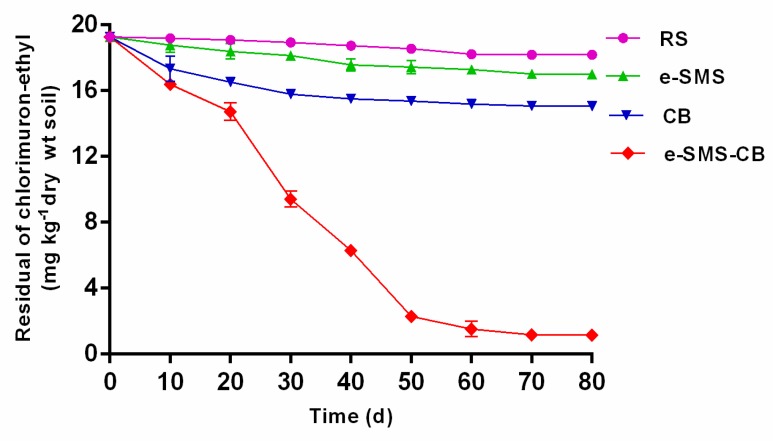
Levels of chlorimuron-ethyl in treated soils. The bars represent the SDs of assays performed in triplicate. RS: real soil as control group; e-SMS: real soil + *P. eryngiu*-SMS; CB: real soil + co-culture bacteria; e-SMS-CB: real soil + *P. eryngiu*-SMS-CB.

After 80 days of incubation, the control group (RS) showed approximately 5.0% degradation of the chlorimuron-ethyl in the chlorimuron-ethyl-contaminated soil, leaving 18.2 mg kg^−1^ residual chlorimuron-ethyl (Figure 2). This result may be related to the abilities of photolysis, chemical hydrolysis and indigenous microorganisms to degrade chlorimuron-ethyl [26]. Inoculation of the soil with *P. eryngiu*-SMS (e-SMS) increased the chlorimuron-ethyl degradation efficiency. After 80 days, the degradation efficiency in the *P. eryngiu*-SMS (e-SMS) group was 10.9%, with 17.1 mg kg^−1^ residual chlorimuron-ethyl. This result may be due to the adsorption of chlorimuron-ethyl by *P. eryngiu*-SMS. A co-culture bacterial solution was added to the chlorimuron-ethyl-contaminated soil (CB), and the degradation efficiency was found to be 21.4%, with 15.1 mg kg^−1^ residual chlorimuron-ethyl after 80 days of incubation. Notably, the degradation efficiency of chlorimuron-ethyl was 80.0% in the MSM group (20 mg L^−1^ chlorimuron-ethyl and cultured at 27 °C after 7 days, with 4.01 mg L^−1^ residual chlorimuron-ethyl). However, the chlorimuron-ethyl residues did not decrease significantly after 30 days, which was most likely due to the bacterial solution becoming inviable in the complex soil environment. Finally, when the *P. eryngiu*-SMS-CB was added to chlorimuron-ethyl-contaminated soil (e-SMS-CB), the chlorimuron-ethyl level slowly decreased slowly during the first 20 days, which may be because the bacteria needed to adapt to the complicated soil environment. During the period from 20 to 50 days, the chlorimuron-ethyl level rapidly decreased from 14.7 mg kg^−1^ to 2.30 mg kg^−1^. The chlorimuron-ethyl level changed slowly from 50 to 80 days, and the overall degradation efficiency was 93.1%, with 1.33 mg kg^−1^ residual chlorimuron-ethyl at 80 days. The above results show that the chlorimuron-ethyl-degrading *P. eryngiu*-SMS-CB has pronounced degradation properties and considerable biodiversity, and it remains stable during long-term treatment in chlorimuron-ethyl-contaminated soil [29].

#### 3.5.3. Bacterial Diversity and Community Structure in Soil

High-throughput sequencing technology has become an established technique in analyzing the distribution of soil microbial community structure [9,30]. Changes in the microbial community composition during the soil remediation processes were determined. The 16S rRNA of soil samples was sequenced using high-throughput sequencing technology. The soil samples collected at 80 days were named Control (80 d), *P. eryngiu*-SMS (80 d) and *P. eryngiu*-SMS-CB (80 d) respectively; the soil samples collected at 180 days were named Control (180 d), *P. eryngiu*-SMS (180 d) and *P. eryngiu*-SMS-CB (180 d), respectively. A total of 425,632 high-quality bacterial V3–V4 Illumina sequences, ranging from 47,988 to 88,183 sequences per read, were obtained for further analysis. The detailed indices are shown in the Table S2.

**Figure 3:**
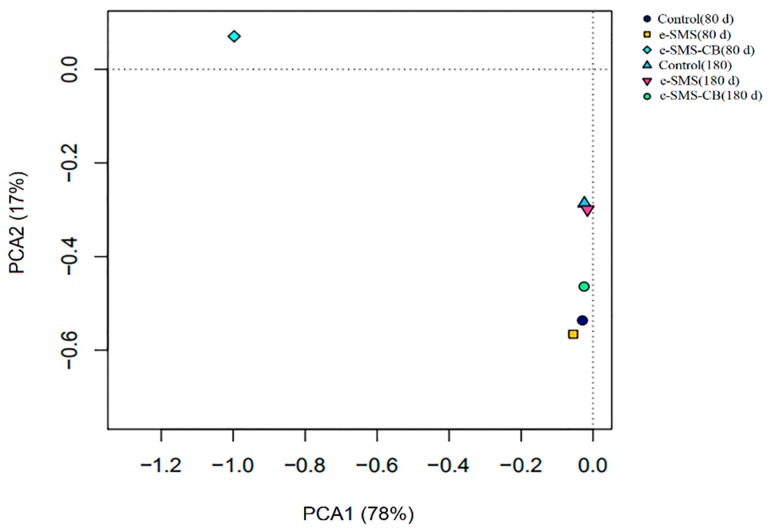
Principal component analysis (PCA) of all six samples based on microbial community composition. Control: real soil as control group; e-SMS: real soil + *P. eryngiu*-SMS; e-SMS-CB: real soil + *P. eryngiu*-SMS-CB.

Principal component analysis (PCA) was used to identify the differences in bacterial community structure between the different experimental groups [31] (Figure 3). As shown in Figure 3, demonstrated that the *P. eryngiu*-SMS-CB (80 d) sample showed a very different position from the other samples, and the Control (180 d) and *P. eryngiu*-SMS (180 d) samples clustered together, separate from to *P. eryngiu*-SMS-CB (180 d). The Control (80 d) and *P. eryngiu*-SMS (80 d) samples grouped together and were distant from *P. eryngiu*-SMS-CB (80 d). These results demonstrated that the bacterial communities in the *P. eryngiu*-SMS-CB (80 d) sample had significant differences from the other samples. The PCA results suggested that the addition of the *P. eryngiu*-SMS-CB greatly changed the soil microbial community structure in a short time. In addition, the soil samples collected at 180 days grouped more closely (similarly) than the soil samples collected at 80 days, thereby indicating the higher number of similar community structures after 180 days [32,33,34]. This finding was consistent with the abovementioned alpha analysis results. Species diversity, which is reflected by the Shannon and Simpson indices, can directly reflect the heterogeneity of a community based on the number of species present and their relative abundances [35]. Higher values of the Shannon index and lower values of the Simpson index indicate high diversity [36]. The species diversity analysis showed that the species diversity of the sample *P. eryngiu*-SMS-CB (80 d) (Shannon index 4.09 and Simpson index 0.15) was lower than those of Control (80 d) (Shannon index 6.54 and Simpson index 0.01) and *P. eryngiu*-SMS (80 d) (Shannon index 6.08 and Simpson index 0.02). Although the species diversity of *P. eryngiu*-SMS-CB (180 d) was still the lowest among the three samples (Control(180 d) (Shannon index 6.89 and Simpson index 5.0e-03), *P. eryngiu*-SMS (180 d) (Shannon index 6.76 and Simpson index 5.3e-03), and *P. eryngiu*-SMS-CB (180 d) (Shannon index 6.50 and Simpson index 9.9e-03)), the differences in sample diversity narrowed as the soil recovery period progressed (shown in Table S2). This result can be explained by the fact that the addition of *P. eryngiu*-SMS-CB into the soil may increase the abundances of certain species, resulting in a decline in sample diversity. For example, *Enterobacter* and *Rhodococcus* were present in the *P. eryngiu*-SMS-CB, and the addition of the *P. eryngiu*-SMS-CB increased the abundance of these two genera, causing them to be dominant bacteria and leading to a decrease in the abundances of other genera at 80 days. As the recovery period progressed and the chlorimuron-ethyl level in the soil decreased after remediation, the abundances of these formerly dominant species decreased, whereas those of the other species increased, and the soil microbial community structure gradually recovered.

**Figure 4:**
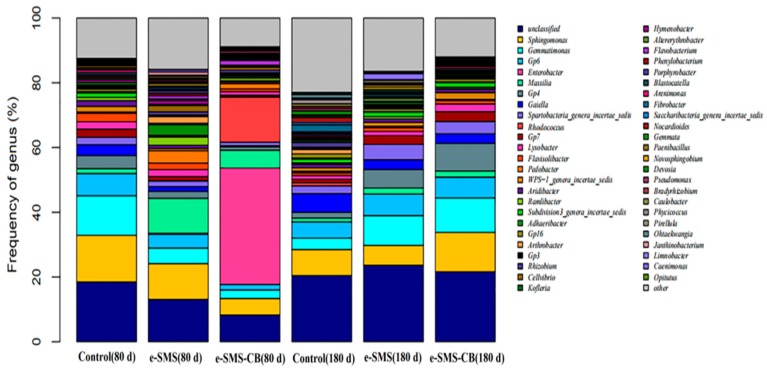
Bacterial community structure in the different samples at the genus level. Organisms with an abundance of less than 1.5% in all samples were classified as “others.” Control: real soil as control group; e-SMS: real soil + *P. eryngiu*-SMS; e-SMS-CB: real soil + *P. eryngiu*-SMS-CB.

After 80 days, at the genus level (Figure 4), the abundances of *Enterobacter* (Control (80 d): 0.01% and *P. eryngiu*-SMS-CB (80 d): 35.9%) and *Rhodococcus* (Control (80 d): 0.02% and *P. eryngiu*-SMS-CB (80 d): 13.9%) were greater in *P. eryngiu*-SMS-CB (80 d) than in Control (80 d). *Enterobacter* and *Rhodococcus* were present in the *P. eryngiu*-SMS-CB. The above results indicated that the addition of *P. eryngiu*-SMS-CB increased the abundance of these two genera, causing them to be dominant bacteria. In addition, the abundances of *Sphingomonas* (Control (80 d): 14.4%, *P. eryngiu*-SMS (80 d): 11.0% and *P. eryngiu*-SMS-CB (80 d): 5.12%), Gp4 (Control (80 d): 4.04%, *P. eryngiu*-SMS (80 d): 2.13% and *P. eryngiu*-SMS-CB (80 d): 0.98%), Gp7 (Control (80 d): 2.53%, *P. eryngiu*-SMS (80 d): 1.24% and *P. eryngiu*-SMS-CB (80 d): 0.60%), *Lysobacter* (Control (80 d): 2.28%, *P. eryngiu*-SMS (80 d): 2.18% and *P. eryngiu*-SMS-CB (80 d): 1.10%) and *Flavisolibacter* (Control (80 d): 2.63%, *P. eryngiu*-SMS (80 d): 2.01% and *P. eryngiu*-SMS-CB (80 d): 0.83%) showed a decreasing trend from Control (80 d) to *P. eryngiu*-SMS (80 d) to *P. eryngiu*-SMS-CB (80 d). *Enterobacter* and *Rhodococcus* were the dominant bacteria and led to a decrease in the abundances of other genera. After 80 days of soil repair, the chlorimuron-ethyl degradation efficiencies in Control (80 d) and *P. eryngiu*-SMS-CB (80 d) were 5.21% and 93.1%, respectively, indicating that the addition of the *P. eryngiu*-SMS-CB promoted the degradation of chlorimuron-ethyl. The abundances of *Massilia* (Control (80 d): 1.46% and *P. eryngiu*-SMS-CB (80 d): 5.51%) and *Pedobacter* (Control (80 d): 0.38%, and *P. eryngiu*-SMS-CB (80 d): 1.66%) in *P. eryngiu*-SMS-CB (80 d) were higher than those in Control (80 d), possibly because the secondary metabolites of the chlorimuron-ethyl degraded by the chlorimuron-ethyl-degrading bacteria could be used by *Massilia* and *Pedobacter*, resulting in increases in their abundances [37]. This result is similar to that of the study by Min, which examined microbial community structure changes in nitrophenol-contaminated soils repaired by degrading bacteria. Increases in the abundances of other genera in soil may result from these genera using secondary metabolites produced by bacterially mediated nitrophenol degradation [38].

Figure 4 shows that the abundances of five genera in *P. eryngiu*-SMS (80 d), *Massilia* (Control (80 d): 1.46% and *P. eryngiu*-SMS (80 d): 10.8%), *Pedobacter* (Control (80 d): 0.38% and *P. eryngiu*-SMS (80 d): 3.81%), *Ramlibacter* (RS: 1.01% and *P. eryngiu*-SMS (80 d): 2.58%), *Adhaeribacter* (Control (80 d): 0.41% and *P. eryngiu*-SMS (80 d): 3.33%), and *Arthrobacter* (Control (80 d): 0.21% and *P. eryngiu*-SMS (80 d): 2.04%), were higher than those in Control (80 d), likely due to the addition of SMS to the soil. SMS may contain nutrients that promote the growth of these genera. This result is similar to that of Garcia-Delgado’s study, which found that the addition of SMS to PAH-contaminated soils can increase the abundance of soil bacteria and fungi in the soil [37].

After 180 days, the abundances of *Enterobacter* (*P. eryngiu*-SMS-CB (80 d): 35.9% and *P. eryngiu*-SMS-CB (180 d): 0.01%) and *Rhodococcus* (*P. eryngiu*-SMS-CB (80 d): 13.9% and *P. eryngiu*-SMS-CB (180 d): 0.02%) were lower in *P. eryngiu*-SMS-CB (180 d) than in *P. eryngiu*-SMS-CB (80 d), indicating that the increased abundances of these genera caused by *P. eryngiu*-SMS-CB decreased with restoration time. Meanwhile, the abundances of other genera were higher in *P. eryngiu*-SMS-CB (180 d) than in *P. eryngiu*-SMS-CB(80 d), for instance, *Sphingomonas* (*P. eryngiu*-SMS-CB (80 d): 5.12% and *P. eryngiu*-SMS-CB (180 d): 12.1%), *Gemmatimonas* (*P. eryngiu*-SMS-CB (80 d): 2.64% and *P. eryngiu*-SMS-CB (180 d): 10.7%), and Gp4 (*P. eryngiu*-SMS-CB (80 d): 0.98% and *P. eryngiu*-SMS-CB (180 d): 8.50%), indicating that the bacteria in the soil that had been affected by *P. eryngiu*-SMS-CB were restored over time. The above results showed that the microbial community structure of the soil changed after 80 days of repair with the *P. eryngiu*-SMS-CB. However, after 180 days of restoration, the microbial community structure had slowly recovered, the chlorimuron-ethyl level in the soil had decreased from 19.2 mg kg^−1^ to 0.38 mg kg^−1^, and the abundances of *Enterobacter* and *Rhodococcus* included in the *P. eryngiu*-SMS-CB had returned to their initial levels. This result can be explained by the fact that the *P. eryngiu*-SMS-CB does not excessively affect the soil microbial community structure in the long term.

#### 3.5.4. Plant Culture

**Figure 5:**
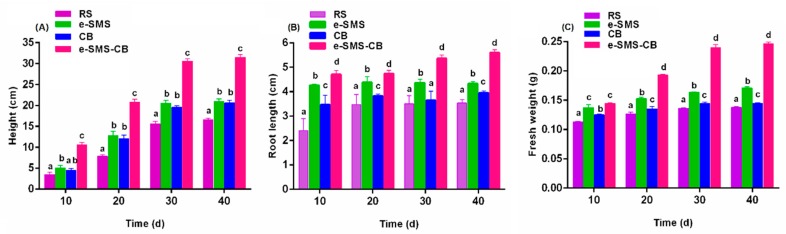
Effects of chlorimuron-ethyl on wheat with increasing incubation time. (**A**) plant height, (**B**) plant length and (**C**) fresh weight. The bars represent the SDs of assays performed in triplicate. RS: real soil as control group; e-SMS: real soil + *P. eryngiu*-SMS; CB: real soil + co-culture bacteria; e-SMS-CB: real soil + *P. eryngiu*-SMS-CB. Bars with different letters are significantly different at the *p* < 0.05 level.

Figure 2 shows the chlorimuron-ethyl residue levels in the soil: RS 18.2 mg kg^−1^, *P. eryngiu*-SMS 17.1 mg kg^−1^, CB 15.1 mg kg^−1^ and *P. eryngiu*-SMS-CB 1.34 mg kg^−1^. The amount of chlorimuron-ethyl residue in the soil significantly affected the height, root length and fresh weight of wheat. As depicted in Figure 5, the chlorimuron-ethyl-contaminated soil was remediated by the *P. eryngiu*-SMS-CB to some extent, and the toxicity of chlorimuron-ethyl toward wheat was reduced.

The growth trends of the wheat in the different groups were similar, although the differences in height, root length, and fresh weight between the groups were significant. The soil was remediated by the *P. eryngiu*-SMS-CB, and the height (31.2 ± 0.25 cm), root length (5.62 ± 0.21 cm) and fresh weight (0.249 ± 0.014 g) of the *P. eryngiu*-SMS-CB treatment were greater than those of the control (RS) (height 16.1 ± 0.25 cm, root length 3.51 ± 0.21 cm and fresh weight 0.146 ± 0.014 g) at 30 days. This finding indicates that the remediation of chlorimuron-ethyl-contaminated soil by the *P. eryngiu*-SMS-CB decreased the stress caused by chlorimuron-ethyl on wheat and promoted its growth. The chlorimuron-ethyl level of the CB treatment (15.1 mg kg^−1^) was lower than that of the *P. eryngiu*-SMS treatment (17.1 mg kg^−1^), but the root length (4.62 ± 0.25 cm) and fresh weight (0.157 ± 0.13 g) were better in *P. eryngiu*-SMS than in CB (root length (3.89 ± 0.07 cm) and fresh weight (0.149 ± 0.009 g)). This was most likely because the *P. eryngiu*-SMS increased soil fertility and promoted wheat growth [39,40].

#### 3.5.5. SOD and SP

Even under natural conditions of growth and development, plants are inevitably exposed to different stress conditions, including chilling, heavy metals, various organic chemicals, and air pollutants. These oxidative stresses generate active oxygen species (AOS) such as hydroxyl (OH^−^) and superoxide (O_2_^−^) radicals, singlet oxygen (^1^O_2_), and hydrogen peroxide (H_2_O_2_), causing tissue injury [41]. Plants have evolved various protective mechanisms to eliminate or reduce the AOS caused by damage and stress. One of these protective mechanisms is the enzymatic antioxidant system, which operates with the sequential and simultaneous actions of several enzymes, including SODs. Changes in SP (soluble protein) content can reflect protein synthesis, denaturation and degradation in cells, and the SP content of plants is reduced under the stresses of chilling, heavy metals, various organic chemicals, and air pollutants. Therefore, changes in SP content and SOD activity may be considered biomarkers of serious stress caused by chlorimuron-ethyl in the soil [4,42].

**Figure 6:**
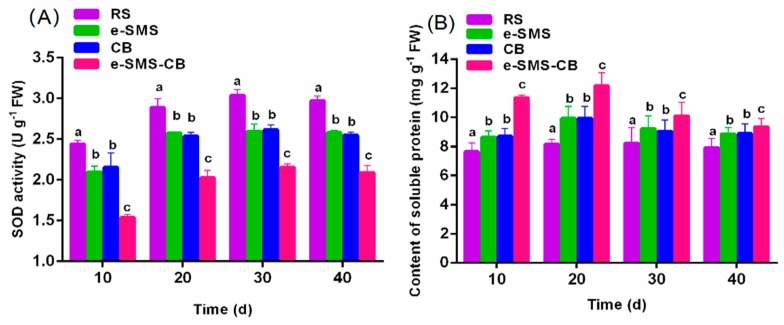
Effects of chlorimuron-ethyl on Superoxide dismutase (SOD) and Soluble protein (SP) of wheat with the increasing incubation time. (**A**) SOD activity and (**B**) SP content. The bars represent the SDs of assays performed in triplicate. Bars with different letters are significantly different at the *p* < 0.05 level. RS: real soil as control group; e-SMS: real soil + *P. eryngiu*-SMS; CB: real soil + co-culture bacteria; e-SMS-CB: real soil + *P. eryngiu*-SMS-CB.

Figure 6A shows that the SOD activity of each experimental group increased with growing time, indicating that chlorimuron-ethyl accumulates in wheat and that increased SOD activity will help to alleviate the oxidative stress caused by chlorimuron-ethyl. The activity of SOD (3.22 ± 0.31 U g^−1^) in RS was significantly higher than that in the *P. eryngiu*-SMS (2.61 ± 0.24 U g^−1^), CB (2.65 ± 0.05 U g^−1^) and *P. eryngiu*-SMS-CB (1.81 ± 0.04 U g^−1^) samples. As illustrated in Figure 6B, the SP content of each group first increased and then stabilized with time after planting. The SP content of *P. eryngiu*-SMS-CB (12.5 ± 0.23 mg g^−1^) was always higher than those of CB (10.0 ± 0.22 mg g^−1^) and *P. eryngiu*-SMS (10.1 ± 0.15 mg g^−1^); however, all these SP contents were significantly higher than that of RS (9.82 ± 0.11 mg g^−1^) at 20 days. The above results show that the toxicity of chlorimuron-ethyl toward wheat was reduced by *P. eryngiu*-SMS-CB.

## 4. Conclusions

In summary, we found that the agricultural waste *P. eryngiu*-SMS, when used as the carrier to prepare *P. eryngiu*-SMS-CB, could effectively support co-culture bacteria. Based on the degradation efficiency and reduced the toxic effects of chlorimuron-ethyl toward wheat planted in treated soil, the *P. eryngiu*-SMS-CB can effectively remediate historically chlorimuron-ethyl-contaminated soil. Our data are in agreement with a number of previous investigations showing that *P. eryngiu*-SMS-CB had a certain degree of impact on the soil bacterial community structure in the short-term, but our in-depth study showed that the *P. eryngiu*-SMS-CB did not affect the soil bacterial community structure in the long term. However, the results obtained in this study were only at the laboratory. Therefore, further studies will be performed to broaden the application range and strengthen the adaptability of the *P. eryngiu*-SMS-CB to the chlorimuron-ethyl-contaminated soil in the field to verify the actual bioremediation effects of the *P. eryngiu*-SMS-CB in situ.

Electronic supplementary data for this work can be found in the online version of the paper.

## Supplementary Materials

The following are available online at https://www.mdpi.com/2076-2607/8/3/369/s1, Figure S1. Effect of pH-values on chlorimuron-ethyl degradation; Figure S2. Effects of culture conditions on the viable counts of the *P. eryngiu*-SMS-CB and the degradation efficiency; Figure S3. Three-dimensional response surface plot; Figure S4. Scanning electron microscopy (SEM) images; Table S1 Variance analysis for the developed quadratic regression model; Table S2 Number of sequences analyzed, OTUs, estimated community richness estimators (Chao and ACE) and community diversity indices (Shannon and Simpson) of 16S rRNA libraries from the samples.
