# Supplementary material for: Bioremediation of Historically Chlorimuron-Ethyl-Contaminated Soil by Co-Culture Chlorimuron-Ethyl-Degrading Bacteria Combined with the Spent Mushroom Substrate
Source: Microorganisms. 2020 Mar 5;8(3):369. doi: 10.3390/microorganisms8030369 (PMC7143799; doi:10.3390/microorganisms8030369)
Supplement: Supplementary file 1 [file microorganisms-08-00369-s001.pdf]

Supplementary Information

Bioremediation of historically chlorimuron-ethyl-contaminated soil by co-culture chlorimuron-ethyl-degrading bacteria combined with the spent mushroom substrate

Hailian Zang[1], Wanjun Liu[1], Yi Cheng[2], Hailan Wang[1], Xuejiao An[3], Shanshan Sun[1], Yue Wang[1], Ning Hou[1], Chunyu Cui[1], Chunyan Li[1]*

[1] College of Resources and Environment, Northeast Agricultural University, Harbin 150030, Heilongjiang, PR China

[2] College of Science, China Agricultural University, Beijing 100083, PR China

[3]College of Bioscience and Bioengineering, Jiangxi Agricultural University, Nanchang 330045, PR China

Materials and methods

The chlorimuron-ethyl extraction assay

For the extraction of chlorimuron-ethyl from the medium, ten milliliters of the culture medium was centrifuged at 12,000 rpm for 10 min, and then 5 mL of the supernatant was transferred to a 250 mL glass separation funnel and extracted with 10 mL of dichloromethane. The supernatants were extracted three times each with 10 mL of dichloromethane, and then the organic phases were combined.

For the extraction of chlorimuron-ethyl from the soil samples, 5 g of soil sample was weighed into a 50 mL polystyrene tube, soaked overnight in 20 mL of dichloromethane and then centrifuged at 12,000 rpm for 10 min. The supernatant was extracted with 10 mL of dichloromethane. Each sample was extracted three times with dichloromethane, and the organic phases were combined.

After dehydration with anhydrous sodium sulfate, the organic phases were

* Corresponding author. Tel.: +86-0451-55190034
E-mail address: chunyanli@neau.edu.cn (Chunyan Li)

collected in a 100 mL flat bottom flask and concentrated to almost dryness with a rotary evaporator (RE52C, Shanghai, China). Acetonitrile was added to dissolve chlorimuron-ethyl, the volume was increased to 1.5 mL, and the samples from all of the biodegradation experiments were filtered through 0.22-μm Millex-GP PES filter for HPLC analysis [1].

HPLC instrumentation and conditions

The chlorimuron-ethyl concentration was determined using a Waters 600 reverse-phase HPLC (Waters, MA, USA) with a Waters 2487 dual wavelength detector and an autosampler with a C18 column (250×4.6 mm i.d., 5 μm particle size). The column temperature was 25°C. The applied mobile phase was methanol/water/glacial acetic acid (70/30/0.5, v/v/v) at a flow rate of 1.0 mL min^{-1}. The injection volume was 20 μL. Ultraviolet absorption was monitored at 254 nm [1, 2]. The peak areas were recorded and calculated using Empower software. The degradation efficiency was calculated with the following formula:

Biodegradation (%) = $(A_0 - A) / A_0 \times 100\%$

where A_0 is the average concentration of the control group, and A is the average concentration of the experimental group.

Extraction of laccase crude extract

A 20 g sample of SMS was placed in an Erlenmeyer flask (250 mL) with 100 mL of sodium citrate buffer (50 mM, pH4.8) and agitated at 150 rpm for 1 h. The crude enzymatic extract was obtained after separation of the residues using filter paper [2]. The crude enzymatic extract was stored in a refrigerator until use.

Assay of Enzymes Activity

Laccase activity was determined by registering the oxidation of 2,2-azo-bis-(ethylbenzothiazoline-6-sulfonic acid) (ABTS) every 20 s during 2 min (λ=420 nm). The reaction mixture included 0.1 mL of crude enzymatic extract, 2.5 mL of sodium acetate buffer (pH 4.5), and 1 mL of ABTS solution (1 mM). The temperature was adjusted to 30°C. One activity unit was defined as the amount of

enzyme needed to oxidize 1 M ABTS per minute.

Effect of pH-values on chlorimuron-ethyl degradation

pH4.0, 5.5, 7.0, 8.5 and 10.0 phosphate buffers was added to MSM supplemented with 20 mg L^{-1} chlorimuron-ethyl, respectively, and then incubated at 27°C for 7 d, and the degradation efficiency of chlorimuron-ethyl was determined.

A 4% (v/v) bacterial suspension was inoculated into MSM supplemented with 20 mg L^{-1} chlorimuron-ethyl, cultured at 27℃ for 7 d. The pH value was measured continuously for 7 d.

A 4% (v/v) bacterial suspension was inoculated into MSM supplemented with 20 mg L^{-1} chlorimuron-ethyl, and the pH of the culture medium was 6.0, 6.5 and 7.0 respectively, cultured at 27℃ for 7 d. Non-inoculated medium was as control group. The degradation efficiency was determined.

Co-culture bacteria

Under aseptic conditions, the chlorimuron-ethyl degrading bacteria *Rhodococcus* sp. D310-1 and *Enterobacter* sp. D310-5 were streaked on LB solid plates and cultured at 30℃ for 48 h, respectively, picking a well-growing single colony on the LB plate, inoculating into LB liquid medium (100 mL, pH 6.5), and incubated at 27°C for about 18 h. After cultured to the logarithmic phase, the culture solution was centrifuged at 5000 rpm for 3 min, the supernatant discarded, and the solution was washed with a phosphate buffer (0.2 mol L^{-1}) for 3 times. The bacteria were collected, and then the concentration of the bacteria was adjusted with a phosphate buffer to OD_{600nm} = 2.0 ± 0.1. Finally, D310-1 and D310-5 were inoculated into the fermentation medium with 2% inoculum, respectively, and the co-culture bacteria were cultured to logarithmic phase (OD=1.92) for subsequent experiments.

Optimization of the conditions for preparing the chlorimuron-ethyl-degrading Pleurotus eryngiu-SMS-CB

The results indicated that chlorimuron-ethyl was degraded by laccase crude extract from SMS, and the SMS of *P. eryngiu* was chosen as a carrier for the

preparation of chlorimuron-ethyl-degrading *P. eryngiu*-SMS-CB.

The *P. eryngiu*-SMS was inoculated with the co-culture bacteria to prepare the *P. eryngiu*-SMS-CB and for optimizing the preparation conditions. The preparation variables included inoculum quantity (2, 2.5, 3, 3.5, 4 and 4.5 mL, OD=1.92), culture time (1, 2, 3, 4 and 5 d), culture temperature (25, 30, 35, 40, 45 and 50°C) and drying temperature (33, 36, 39, 42 and 45°C). The prepared *P. eryngiu*-SMS-CB was added to MSM supplemented with 20 mg L^{-1} chlorimuron-ethyl. The optimal preparation conditions were determined using the chlorimuron-ethyl degradation efficiency and the count of living cells as indices.

Based on preliminary experiments, a Box–Behnken Design (BBD) was used to optimize *P. eryngiu*-SMS-CB preparation conditions. A second-order polynomial equation was a typical model that could describe the response surface:

$$Y = A + \sum_{j=1}^{k} A_j X_j + \sum_{j=1}^{k} A_{jj} X_j^2 + \sum_{i} \sum_{<j=2}^{k} A_{ij} X_i X_j$$

where Y is the predicted response; A is a constant and A_j, A_{jj} and A_{ij} are coefficients of the linear, square and interaction terms, respectively.

To validate the predicted results with the optimized model, the degradation efficiency was measured in triplicate under optimum conditions.

Results and discussion

Effect of pH-values on chlorimuron-ethyl degradation

In general, sulfonylurea herbicides exist as a mixture of ions and molecules in the environment. Under acidic conditions, sulfonylurea herbicides are easily hydrolyzed. Under alkali conditions, the negative charge is distributed on the sulfonylurea bridge, which reduces the nucleophilic reactivity of the carbonyl carbon atoms, and these herbicides are not easily hydrolyzed[4]. Therefore, the hydrolysis of sulfonylurea herbicides is affected by pH. In this study, the effects of pH on chlorimuron-ethyl hydrolysis were examined, and the results are shown in Fig. S1a. The chlorimuron-ethyl hydrolysis efficiency decreased with increasing pH. The hydrolysis efficiency reached 40.5% (pH=4.0), 21.3% (pH=5.5), 10.7% (pH=7.0), 10.5% (pH=8.5), and 4.23% (pH=10) after 7 d, respectively. Therefore, chlorimuron-ethyl was easily hydrolyzed under acidic conditions. Changes in pH were observed during the degradation of chlorimuron-ethyl by strains D310-1 and

D310-5, and the results are shown in Fig. S1b. The medium pH increased from 6.5 to 7.0 after strains D310-1 and D310-5 were cultured continuously for 7 d, and the pH of the control group remained at the initial pH of 6.5. At different pH values (6.0, 6.5 and 7.0) of the culture medium, the chlorimuron-ethyl degradation efficiencies by D310-1 and D310-5 reached 80.7%, 84.5%, 81.5% after 7 d, respectively, and the hydrolysis efficiencies were only 11.3%, 10.7%, and 10.1%, respectively (Fig. S1c). During the degradation of chlorimuron-ethyl, the pH of the culture medium did not change significantly, so the degradation of chlorimuron-ethyl was mainly based on microbial degradation, not an acidolysis.

Optimization of the preparation conditions for the P. eryngiu-SMS-CB using RSM

Fig. S2a indicates that the chlorimuron-ethyl degradation efficiency of the *P. eryngiu*-SMS-CB peaked at 35℃. Fig. S2b shows that the number of live bacteria and the chlorimuron-ethyl degradation efficiency peaked at 4 d. In Fig. S2c, the optimal inoculum quantity was 3.0 mL. The maximum chlorimuron-ethyl degradation efficiency and the highest bacterial yield were 92.6% and 1.69×10^{10} CFU g^{-1}, respectively. As shown in Fig. S2d, both the counts of live bacteria and the chlorimuron-ethyl degradation efficiency were stable and exhibited no obvious change at drying temperatures of 30-38℃; however, they decreased at the increased temperatures of 42 and 46℃. Different drying temperatures (30, 34 and 38℃) for the BC required different times (4, 3 and 2 d, respectively) for complete drying. From an economic point of view and for a reduction in production time, 38℃ was chosen as the optimal drying temperature.

Based on preliminary experiments, all of the variables, inoculum quantity (2, 3, and 4 mL), the culture time (2, 3, and 4 d), and the culture temperature (30, 35, and 40℃), were fixed at 3 levels (−1, 0, 1). A quadratic model of Box–Behnken Design was selected. A total of seventeen runs were conducted to obtain the best levels of variables. Three parameters (inoculum quantity, culture time, and culture temperature) were taken as independent variables, and the chlorimuron-ethyl degradation efficiency was taken as the response of the study. The equation is shown as follows:

$$Y=94.35-2.76A-0.69B+2.73C-1.91AB+2.71AC+1.82BC-8.87A^2-5.49B^2-7.72C^2$$

where Y is the predicted degradation efficiency of chlorimuron-ethyl, A is the inoculum quantity, B is the culture time and C is the culture temperature.

The ANOVA for the second-order polynomial model is shown in Table S1. The regression model, with an R^2=0.9981, was considered to exhibit very high correlation. The closer the value of the correlation coefficient is to 1, the better the correlation is between the experimental values and predicted ones. An Adeq Precision value of the model greater than 4 indicates that the model is reasonable, and since the model Adeq Precisior1=62.778, the model is reasonable. The regression models were statistically acceptable, with *P*-values (0.5200) > 0.05 (not significantly different) and an *F*-value of 406.47. Table S1 shows that A, B, C, AB, BC, AC, A^2, B^2 and C^2 were significant terms ($p < 0.05$), indicating that the independent variables and interactive variables have a significant effect on the chlorimuron-ethyl degradation efficiency of *P. eryngiu*-SMS-CB.

In the response analysis experiment, the *p*-value of the independent variables culture time (A), inoculating liquid quantity (B), and culture temperature (C) were < 0.0001, 0.0067, < 0.0001, respectively, indicating that independent variables have a significant effect on the chlorimuron-ethyl degradation efficiency of *P. eryngiu*-SMS-CB. The BC term related to culture time (*F*=230.45) and culture temperature (*F*=225.47) showed that culture time has a significant effect.

The *P*-values of the interaction variables AB, AC and BC were 0.0001, <0.0001 and 0.0002, respectively, all less than 0.01, indicating that the interaction variables of AB, AC and BC have an extremely significant effect on the chlorimuron-ethyl degradation efficiency of *P. eryngiu*-SMS-CB. The *p*-value of AC was the smallest, indicating that AC has a more significant impact on the chlorimuron-ethyl degradation efficiency of *P. eryngiu*-SMS-CB than the AB and BC terms.

RSM was used to graphically represent the effects of the independent variables (culture time, inoculum quantity, culture temperature) and their interaction on the chlorimuron-ethyl degradation efficiency of *P. eryngiu*-SMS-CB by three-dimensional response surface plots (Fig. S3). Each figure shows the effect of 2 factors with the third factor is set at central levels. Fig. S3a indicates how the interaction of the inoculum quantity and the culture time affected the chlorimuron-ethyl degradation efficiency. When the culture temperature was fixed at central levels (35°C), the degradation efficiency of chlorimuron-ethyl increased slightly as the inoculum quantity and culture time increased until the maximum degradation efficiency was achieved. The degradation efficiency decreased with increasing inoculum quantity and culture time. Fig. S3b describes how interactions

between culture temperature and culture time affected the chlorimuron-ethyl degradation efficiency of *P. eryngiu*-SMS-CB. The culture time and culture temperature increased while the inoculum quantity was fixed at central levels (3 mL), and the chlorimuron-ethyl degradation efficiency of the *P. eryngiu*-SMS-CB increased and subsequently decreased. Fig. S3c shows the effects of the culture temperature and inoculum quantity on the chlorimuron-ethyl degradation efficiency of *P. eryngiu*-SMS-CB when the culture time was maintained. In contrast to Fig. S3a and Fig. S3c, Fig. S3b shows that the interaction between culture time and culture temperature has a more significant effect on the degradation of chlorimuron-ethyl by *P. eryngiu*-SMS-CB than the other interactions. Use of the model and Design Expert 8.0 predicted that the best values of the 3 key factors were an inoculum quantity of 2.99 mL, a culture temperature of 35.7°C and a culture time of 2.87 d. Under the above conditions, the highest degradation efficiency of *P. eryngiu*-SMS-CB was 94.4%.

References

[1] Li, C.Y.; Zang, H.L.; Yu, Q.; Lv, T.Y.; Cheng, Y.; Cheng, X.S.; Liu, K.R.; Liu, W.J.; Xu, P.P.; Lan, C.Z. Biodegradation of chlorimuron-ethyl and the associated degradation pathway by *Rhodococcus* sp. D310-1. *Environ. Sci. Pollut. Res.* 2016, 23, 8794-8805.

[2] Zang, H.L.; Wang, H.L.; Miao, L.; Cheng, Y.; Zhang, Y.T.; Liu, Y.; Sun, S.S.; Wang, Y.; Li C.Y. Carboxylesterase, a de-esterification enzyme, catalyzes the degradation of chlorimuron-ethyl in *Rhodococcus erythropolis* D310-1, *J. Hazard. Mater.* 2019, 121684.

[3] Luz, J.M.R.; Nunes, M.D.; Paes, S.A.; Torres, D.P.; Da Silva, M.D.S. Lignocellulolytic enzyme production of *Pleurotus ostreatus* growth in agroindustrial wastes. *Braz. J. Microbiol.* 2012, 43, 1508-1515.

[4] Braschi, I.; Pusino, A.; Gessa, C.; Bollag, J.M. Degradation of primisulfuron by a combination of chemical and microbiological processes. J. Agr. *Food Chem.* 2000, 48, 2565-71.

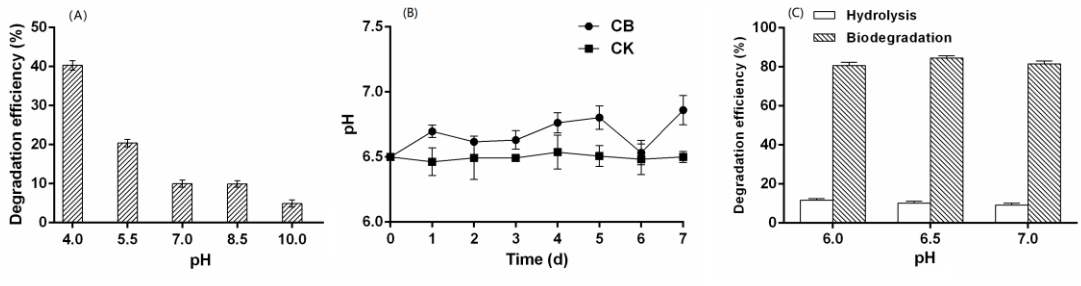

Fig. S1. Effect of pH-values on chlorimuron-ethyl degradation. The bars represent the SDs of assays performed in triplicate. (A) Effects of pH on the hydrolysis degradation of chlorimuron-ethyl; (B) Changes in pH by chlorimuron-ethyl-degrading bacteria; (C) Effect of pH on degradation of chlormuron-ethly by bacteia.

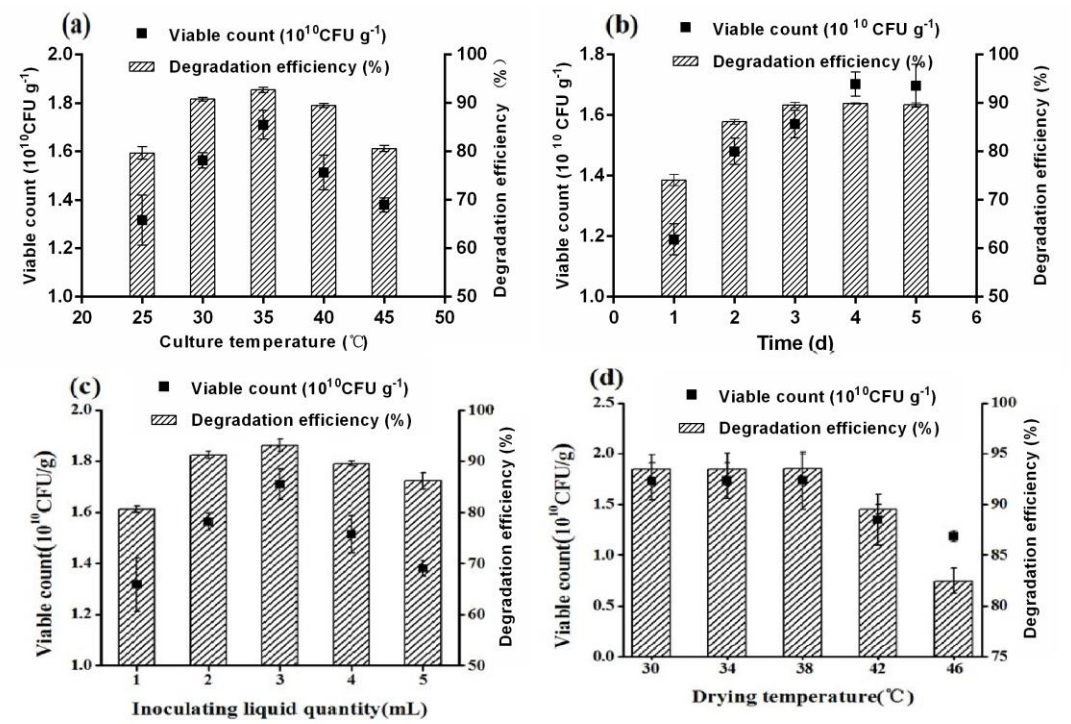

Fig. S2. Effects of culture conditions on the viable counts of the *P. eryngiu*-SMS-CB and the degradation efficiency (a) culture temperature; (b) culture time; (c) inoculum liquid quantity; (d) drying temperature. The bars represent the SDs of assays performed in triplicate.

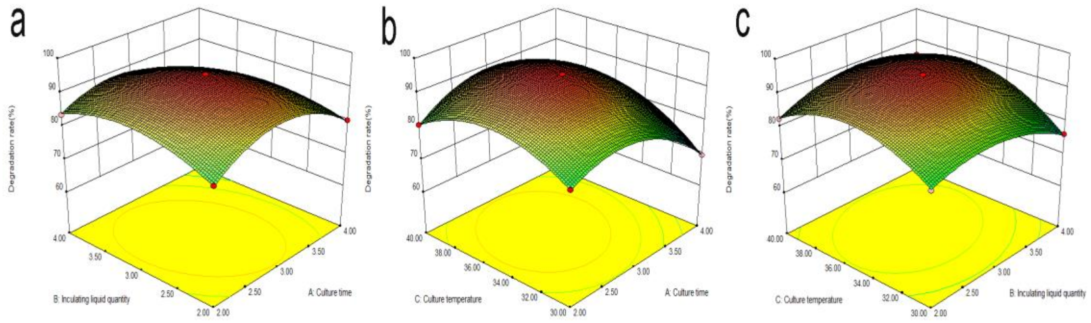

Fig. S3. Three-dimensional response surface plot for (a) the interaction effect of inoculating liquid quantity and culture time; (b) the interaction effect of culture time and culture temperature; (c) the interaction effect of inoculating liquid quantity and culture temperature on the degradation efficiency of *P. eryngiu*-SMS-CB

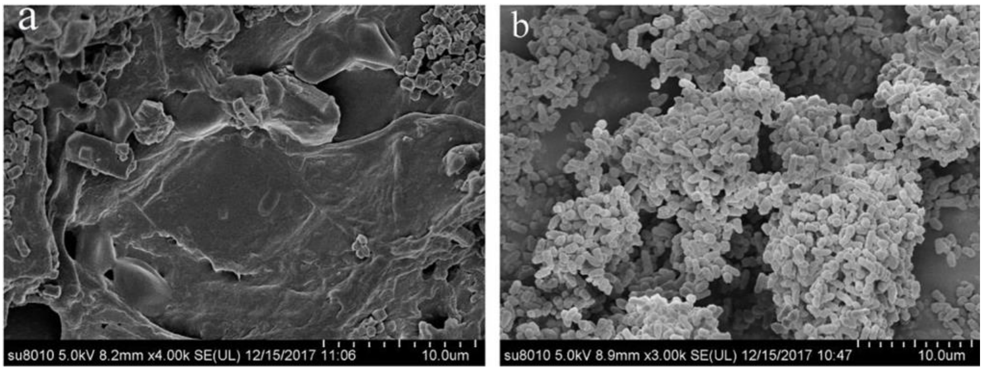

Fig. S4. Scanning electron microscopy (SEM) images of the empty carrier (a) and the co-culture bacteria (b).

Table S1 Variance analysis for the developed quadratic regression model

Source	SS	df	Mean square	*F*-value	*P*-value	
Model	970.03	9	107.78	406.47	< 0.0001	significant
A	61.11	1	61.11	230.45	< 0.0001	
B	3.84	1	3.84	14.47	0.0067	
C	59.79	1	59.79	225.47	< 0.0001	
AB	14.63	1	14.63	55.18	0.0001	
AC	29.27	1	29.27	110.38	< 0.0001	
BC	13.21	1	13.21	49.83	0.0002	
A^2	331.12	1	331.12	1248.75	< 0.0001	
B^2	126.70	1	126.70	477.81	< 0.0001	
C^2	251.14	1	251.14	947.10	< 0.0001	
Residual	1.86	7	0.27			
Lack of Fit	0.74	3	0.25	0.89	0.5200	not significant
Pure Error	1.11	4	0.28			
Cor Total	971.88	16				
R^2	0.9981					
R^2_{Adj}						
Pred	0.9956					
R-Squared	0.9860					
Adeq	62.778					
Precision						

Table S2 Number of sequences analyzed, OTUs, estimated community richness estimators (Chao and ACE) and community diversity indices (Shannon and Simpson) of 16S rRNA libraries from the samples

Sample ID	Seq_num	OTU_num	Shannon	Ace	Chao1	Simpson
Control(80 d)	88183	5830	6.54	7789.73	7421.96	0.01
P.eryngiu-SMS-CB(80 d)	53284	3063	4.09	5747.42	4570.76	0.15
P.eryngiu-SMS(80 d)	80932	5030	6.08	7233.23	6794.45	0.02
Control(180 d)	78700	5711	6.89	7422.59	7130.34	5.0e-03
P.eryngiu-SMS-CB(180 d)	47988	4503	6.50	6501.74	6224.20	9.9e-03
P.eryngiu-SMS(180 d)	76545	5639	6.76	7720.80	7530.64	5.3e-03

A total 425632 high-quality bacterial V3–V4 Illumina sequences, ranging from 47988 to 88183 sequences per read, were obtained for further analysis. The richness of Control(80 d) (ACE index 7789.73 and Chao1 index 7421.96) was higher than that of *P.eryngiu*-SMS(80 d) (ACE index 7233.23 and Chao1 index 6794.45), and they values of both were higher than that of *P.eryngiu*-SMS-CB(80 d) (ACE index 5747.42 and Chao1 index4570.76). The richness of the sample *P.eryngiu*-SMS(180 d) (ACE index 7720.80 and Chao1 index 7530.64) was higher than that of Control(180 d) (ACE index 7422.59 and Chao1 index 7130.34), and the values of both were higher than that of *P.eryngiu*-SMS-CB(180 d) (ACE index 6501.74 and Chao1 index 6224.20).

The species diversity of the sample Control(80 d) (Shannon index 6.54 and Simpson index 0.01) was higher than that of *P.eryngiu*-SMS(80 d) (Shannon index 6.08 and Simpson index 0.02), with both indices being higher than that of *P.eryngiu*-SMS-CB(80 d) (Shannon index 4.09 and Simpson index 0.15). The species diversity of Control(180 d) (Shannon index 6.89 and Simpson index 5.0e-03) was higher than that of *P.eryngiu*-SMS(180 d) (Shannon index 6.76 and Simpson index 5.3e-03), with both indices being higher than that of *P.eryngiu*-SMS-CB(180 d) (Shannon index 6.50 and Simpson index 9.9e-03) at 180 d.
